# Spatial-Temporal Effects of PM_2.5_ on Health Burden: Evidence from China

**DOI:** 10.3390/ijerph16234695

**Published:** 2019-11-25

**Authors:** Ming Zeng, Jiang Du, Weike Zhang

**Affiliations:** 1 School of Economics, Sichuan University, Chengdu 610065, China; zengming951124@163.com (M.Z.); dujiang_1@163.com (J.D.); 2 School of Public Administration, Sichuan University, Chengdu 610065, China; 3 Andalusian Research Institute in Data Science and Computational Intelligence (DaSCI), University of Granada, 18071 Granada, Spain

**Keywords:** PM_2.5_ exposure, health burden, spatial spillover effect, spatial Durbin model (SDM)

## Abstract

By collecting the panel data of 29 regions in China from 2008 to 2017, this study used the spatial Durbin model (SDM) to explore the spatial effect of PM_2.5_ exposure on the health burden of residents. The most obvious findings to emerge from this study are that: health burden and PM_2.5_ exposure are not randomly distributed over different regions in China, but have obvious spatial correlation and spatial clustering characteristics. The maximum PM_2.5_ concentrations have a significant positive effect on outpatient expense and outpatient visits of residents in the current period, and the impact of PM_2.5_ pollution has a significant temporal lag effect on residents’ health burden. PM_2.5_ exposure has a spatial spillover effect on the health burden of residents, and the PM_2.5_ concentrations in the surrounding regions or geographically close regions have a positive influence on the health burden in the particular region. The impact of PM_2.5_ exposure is divided into the direct effect and the indirect effect (the spatial spillover effect), and the spatial spillover effect is greater than that of the direct effect. Therefore, we conclude that PM_2.5_ exposure has a spatial spillover effect and temporal lag effect on the health burden of residents, and strict regulatory policies are needed to mitigate the health burden caused by air pollution.

## 1. Introduction

With the rapid development of China’s economy, the living standard and health of residents have been greatly improved, but ambient air pollution remains a serious problem. The Global Burden of Disease (GBD) and the World Bank ranked air pollution as the fifth and fourth global health risk factor, respectively (Global Burden of Disease (GBD), 2015; World Bank, 2016). Air pollution is associated with increased incidence of diseases (WHO, 2012). As air pollution is one of causes of death and disability, it is increasingly recognized as a worldwide public health concern [1,2,3,4,5,6,7]. Atmospheric particle matter (PM), especially those having an aerodynamic diameter less than 2.5 um (PM_2.5_), is considered one of the priority pollutants in air [8,9,10]. As the smog worsened, PM_2.5_ pollution became the main air pollutant in China and played a non-negligible role in affecting Chinese residents’ health [11,12,13]. For example, in 2018, only 35.8% of 338 cities satisfied air-quality standards in China, while the rest of the cities exceeded them. In 2018, all the 338 cities experienced on average 8.05 days with severe air pollution, and the average annual PM_2.5_ concentrations in 56.20% of the cities exceeded 35.74 μg/m^3^. Also, of the 169 key cities, 140 cities had PM_2.5_ as the main pollutant, and 30% cities had an air quality index (AQI) greater than 100 (Air quality index (AQI) is a quantitative description of air quality. The larger the value is, the more serious the air pollution is, and the more harmful it will be to human health. The main pollutants for air quality evaluation are particulate matter (PM_2.5_), inhalable particulate matter (PM_10_), sulfur dioxide (SO_2_), nitrogen dioxide (NO_2_), ozone (O_3_) and carbon monoxide (CO)) [14]. 

PM_2.5_ often contains heavy metals such as arsenic, chromium, and manganese, and PM_2.5_ concentrations in the region mainly depend on its energy efficiency [15,16,17,18]. Frequent exposure to PM_2.5_ can cause great damage to human health, and then leads to the increment of patient visits and health expense, which brings great economic burden to residents. According to China’s National Statistics Bureau (CSB), the total health expenditure increased from 359.39 billion RMB in 2008 to 1639.91 billion RMB in 2018, with an average annual growth rate of 18.73% (CSB, 2018). Share of health expenditure increased from 4.55% in 2008 to 6.57% in 2018 (Share of health expenditure refers to the ratio of total health expenditure to gross domestic product (GDP)). Per capita health expenditure rose from 1094.5 RMB in 2008 to 4237 RMB in 2018, and its average annual growth rate was 15.49% (Per capita health expenditure refers to the ratio of total health expenditure to the total population.). Besides, per capita outpatient visits increased from 3.7 times in 2008 to 6.0 times in 2018 (Per capita outpatient visits refers to the ratio of total number of outpatient visits to the total population) [19]. This suggests that the Chinese government is increasingly investing in health expenditure to improve public health and make or become less the burden of residents.

In recent years, there has been an increasing amount of literature on PM_2.5_ exposure and health [20,21]. Several studies have explained that PM_2.5_ exposure can cause many kinds of diseases, such as respiratory diseases [22,23,24,25], cardiopulmonary diseases [26,27], cardiovascular diseases [24,28], lung cancer [25,27,29,30], and brain damage [31,32,33,34], and it is even closely related to mortality [35]. Although studies have directly or indirectly proved the health effects of PM_2.5_ exposure, conclusions are inconsistent for the differences in the sample selection or data characteristics (such as time series, panel data, experimental data, etc). Some studies found that PM_2.5_ exposure can harm the residents’ health, reduce their labor capacity and shorten their life expectancy, thus further increasing health expenses, and imposing huge economic burden on the whole society [36,37,38,39]. For example, Yang et al. [37] found that the economic loss caused by PM_2.5_ pollution in Beijing in 2013 was 1.11 billion RMB. Zeng et al. [40] used the spatial interpolation method to explore the PM_2.5_ exposure in China in 2007, and found that economic loss was 1,262.5 billion RMB. Although some studies have begun to explore the impact of PM_2.5_ exposure on economic losses, few studies analyze the PM_2.5_ exposure on health burden in detail.

Moreover, with the unprecedented economic development and urbanization in recent decades in China, energy consumption has increased significantly and PM_2.5_ pollution has become a serious problem [41]. Because of the different economic development and urbanization in different regions in China, PM_2.5_ pollution varies significantly in different regions. More importantly, different regions are not independent with each other, and some phenomena in one region are closely related to the same phenomena in other regions [42,43,44,45]. From here, it is necessary to take the spatial correlation and spatial spillover effects into account when analyzing the impact of PM_2.5_ on health burden. If the study ignores the spatial effect, the conclusion may not be accurate. However, exiting studies have rarely considered the spatial spillover effect of PM_2.5_ exposure on health burden [46,47,48].To fill the gaps mentioned above, we devoted this study to explore the impact of PM_2.5_ exposure on the health burden from the perspective of spatial spillover effect. The main novelties and contributions of this paper were listed as follows:

(1) This study explored the impact of PM_2.5_ exposure on the residents’ health burden, further enriching the research perspective of economic loss brought by air pollution.

(2) The spatial econometric models were applied to examine the spatial spillover effects of PM_2.5_ exposure on residents’ health burden, and its spatial dependence and correlation were also discussed.

(3) The temporal lag effect was investigated in this study. Besides, outpatient expense and outpatient visits were presented to measure the health burden, as well as the number of hospitalizations was selected to test robustness.

## 2. Research Design

### 2.1. Data

This study focused on the spatial impact of PM_2.5_ exposure on health burden. We selected 29 regions in China from 2008 to 2017 as samples. These regions including: Beijing, Tianjin, Hebei, Shanxi, Inner Mongolia, Liaoning, Jilin, Heilongjiang, Shanghai, Jiangsu, Zhejiang, Anhui, Fujian, Jiangxi, Shandong, Henan, Hubei, Hunan, Guangdong, Guangxi, Chongqing, Sichuan, Guizhou, Yunnan, Shaanxi, Gansu, Qinghai, Ningxia and Xinjiang. As China only began releasing the ground-level PM_2.5_ concentrations data in 2013, this study adopted the long-term data published by Columbia University Center for Socio-Economic Data and Applications [49]. The PM_2.5_ concentration data in different regions of China is aerosol optical depth (AOD) data from satellite retrieval of surface PM_2.5_ concentration retrievals. The other indicator data were collected from the Chinese Health Statistics Yearbook 2008-2017 [50], including outpatient expense, outpatient visits, the number of hospitalizations, the number of medical institutions, the number of hospital beds and the number of doctors. The data of GDP and the ratio of urban population was from China Statistics Yearbook 2008–2017 [51].

### 2.2. Variable

#### 2.2.1. Dependent Variable: Health Burden

Most existing studies only adopted the number of outpatient visits to measure the health burden of residents [52,53]. But it cannot accurately measure the health burden of residents since the results may be biased [54,55]. Also, some literature used the number of hospitalization to measure health burden [56,57]. However, Chinese residents have a habit of not seeking medical treatment if they are not seriously ill, and the number of hospitalization used may underestimate the health burden. Therefore, to ensure the reliability of the results, this study measured health burden using outpatient expense (exp_out) and outpatient visits (num_out). Outpatient expense is expressed as the ratio of the total outpatient expense to the total number of outpatient visits, and outpatient visits are expressed as the ratio of the total number of outpatient visits to the total population. Besides, a robustness test was performed by using the number of hospitalizations (num_hos) which is expressed as the ratio of the total number of hospitalizations to the total population.

#### 2.2.2. Independent Variable: PM_2.5_ Exposure

PM_2.5_ exposure has a significant impact on the health burden of residents. This study utilized the PM_2.5_ concentration data to analyze the spatial impact of PM_2.5_ exposure on health burden. In fact, the accurate estimation of PM_2.5_ concentrations is one of the most critical preconditions. This study adopted the PM_2.5_ data in 2007–2017 published by Columbia University (The average PM_2.5_ concentrations and the maximum PM_2.5_ concentrations the of 29 regions in China from 2007 to 2017 are listed in Table A1 and Table A2). Some literature used average PM_2.5_ concentrations to measure PM_2.5_ exposure [48,58]. However, severe air pollution, such as the maximum concentrations of PM_2.5_, may cause more harm to residents’ health. Therefore, this study used maximum PM_2.5_ concentrations (PM_2.5_max_) to measure PM_2.5_ exposure. Meanwhile, due to the temporal lag effect of PM_2.5_ exposure on residents’ health burden [48], this study also used the maximum of PM_2.5_ concentrations lags by one stage (PM_2.5_max_(-1)) as the independent variable to verify whether the temporal lag effect exists. In addition, average PM_2.5_ concentrations (PM_2.5_avg_) was used as a substitute variable for PM_2.5_ exposure to test the robustness of the results.

#### 2.2.3. Control Variable

As all know, health burden is affected not only by PM_2.5_ exposure, but also by many others. Referring to the existing studies [30,48], this study controlled the following variables: per capita gross domestic product (PGDP), the ratio of urban population (urban), the number of medical institutions (num_inst), the number of hospital beds (num_bed) and the number of doctors (num_doctor).

The variables and their definitions were presented in Table 1, and the descriptive statistics of all were shown in Table 2.

### 2.3. Method

#### 2.3.1. Spatial Autocorrelation Test

To comprehensively explore the spatial spillover effect of PM_2.5_ exposure, we used the global and the local spatial correlation indices in Exploratory Spatial Data Analysis (ESDA) [59] to test the spatial correlation. The global and the local spatial correlation indices were measured by Moran’s index (Moran’s I), and their calculation formulas were shown as follows:

(1) $$I=\frac{\sum_{i=1}^{n}\sum_{j=1}^{n}w_{ij}(x_{i}-\bar{x})(x_{j}-\bar{x})}{S^{2}\sum_{i=1}^{n}\sum_{j=1}^{n}w_{ij}},\bar{x}=\frac{1}{n}\sum_{i=1}^{n}x_{i},S^{2}=\frac{1}{n}{\sum_{i=1}^{n}\left(x_{i}-\bar{x}\right)}^{2}$$

(2) $$I_{i}=\frac{\left(x_{i}-\bar{x}\right)}{S^{2}}\sum_{j=1}^{n}w_{ij}(x_{j}-\bar{x}),\bar{x}=\frac{1}{n}\sum_{i=1}^{n}x_{i},S^{2}=\frac{1}{n}{\sum_{i=1}^{n}\left(x_{i}-\bar{x}\right)}^{2}$$

where, x_i_ and x_j_ represent the observed values of region i and region j, respectively; n represents the number of all regions; w_ij_ is the element in the spatial weight matri x; $\bar{x}$ is the mean value of the sample, and S^2^ is the variance of the sample.

For Moran’s I, its value range is [−1,1]. If its value is greater than 0, it indicates that there is a positive spatial correlation among variables. If its value is smaller than 0, it indicates that there is a negative spatial correlation among variables. Meanwhile, the values of Moran’s I in different regions can be plotted as scatter plots to view the degree of spatial agglomeration in the regions. The slope of the regression line of the scatter plot is equal to the value of global Moran’s I.

In the spatial econometric analysis, it is necessary to introduce spatial weighting matrices to describe the relationship among different regions. To systematically explore the spatial correlation characteristics among different regions in China, this study modeled the following three spatial weight matrices: spatial contiguity matrix W_1_, spatial distance matrix W_2_, and spatial economy matrix W_3_.

The spatial contiguity matrix is most widely used in spatial econometric analysis, but sometimes the relationship among regions is so simplified that research conclusions are biased. The elements of spatial contiguity matrix W_1_ were defined as follows [47]:

(3) $$w_{ij}=\{\begin{array}{c} 1,i\neq j\\ 0,i=j \end{array}\;\;\;\;i,j=1,2,\cdots ,n$$

To enhance the robustness of the analysis results, we also constructed the spatial distance matrix. The elements of spatial distance matrix W_2_ were defined as follows [47]:

(4) $$w_{ij}=\{\begin{array}{l} 1/d_{ij},i\neq j\\ 0,i\neq j \end{array}\;\;\;\;i,j=1,2,\cdots ,n$$

where d_ij_ represents road distance between region i and region j.

The spatial contiguity matrix and spatial distance matrix only reflect the influence of geographical location, but do not reflect the economic correlation among regions and their influence. For example, the influence of Hebei province on Beijing municipal is much smaller than that of Beijing municipal on Hebei province. Referring to the related studies [47], this study defined the spatial economy matrix W_3_ as follows:

(5) $$\begin{array}{l} \begin{array}{c} W_{3}=W_{1}*\frac{1}{\bar{Y}}diag(\bar{Y_{1}},\bar{Y_{2}},\cdots ,\bar{Y_{n}})\\ \bar{Y_{i}}=\frac{1}{t_{1}-t_{0}+1}\sum_{t=t_{0}}^{t_{1}}Y_{it},\bar{Y}=\frac{1}{n}\sum_{i=1}^{n}\bar{Y_{i}} \end{array}\;i,j=1,2,\cdots ,n \end{array}$$

where Y_it_ is the per capita real GDP of region i in year t; $\bar{Y_{i}}$ represents the annual average of per capita real GDP of region i. Y is the average of $\bar{Y_{i}}$ for all the regions. As can be seen from the formula (5), if the per capita real GDP of region i is greater than that of other regions, the region also has more influence on the other regions.

#### 2.3.2. Spatial Econometric Model

This study used spatial econometric models to analyze the impact of PM_2.5_ exposure on residents’ health burden in China, and to measure the direct effect and the spatial spillover effect. Three widely used spatial econometric models were adopted to examine the spatial effects, including the spatial autoregression model (SAR), spatial errors model (SEM) and spatial Durbin model (SDM) [60]. SAR only includes the lag term of the spatial dependent variable, and SEM only includes spatial spillover effects of independent variables, while SDM includes both the lag term of the spatial dependent variable and spatial spillover effects of independent variables. Based on this, these three spatial econometric models were constructed as follows:

SAR:

(6) $$\begin{array}{l} Y_{it}=\alpha \;+\;\rho W*Y_{it}+\beta_{1}PM_{2.5}\_max_{it}+\beta_{2}PM_{2.5}\_max{\left(-1\right)}_{it}\\ \;\;\;\;\;\;\;\;+\beta_{3}PGDP_{it}+\beta_{4}urban_{it}+\beta_{5}num\_inst_{it}\\ \;\;\;\;\;\;\;\;+\beta_{6}num\_bed_{it}+\beta_{7}num\_doctor_{it}+\varepsilon_{it} \end{array}$$

SEM:

(7) $$\begin{array}{l} Y_{it}\;=\;\alpha +\beta_{1}PM_{2.5}\_max_{it}+\beta_{2}PM_{2.5}\_max{\left(-1\right)}_{it}\\ \;\;\;\;\;\;\;\;+\beta_{3}PGDP_{it}+\beta_{4}urban_{it}+\beta_{5}num\_inst_{it}\\ \;\;\;\;\;\;\;\;+\beta_{6}num\_bed_{it}+\beta_{7}num\_doctor_{it}+u_{it}\\ u_{it}=\lambda Wu_{it}+\varepsilon_{it},\;\varepsilon \sim N(0,\sigma^{2}I_{n}) \end{array}$$

SDM:

(8) $$\begin{array}{l} Y_{it}\;=\;\alpha +\rho W*Y_{it}+\beta_{1}PM_{2.5}\_max_{it}+\beta_{1}PM_{2.5}\_max{\left(-1\right)}_{it}\\ \;\;\;\;\;\;\;\;+\beta_{3}PGDP_{it}+\beta_{4}urban_{it}+\beta_{5}num\_inst_{it}+\beta_{6}num\_bed_{it}\\ \;\;\;\;\;\;\;\;+\beta_{7}num\_doctor_{it}+\sigma W*X_{kit}+\varepsilon_{it} \end{array}$$

where Y is the dependent variable; PM_2.5_max_ and PM_2.5_max_(−1) are the core independent variable; PGDP, urban, num_inst, num_bed, and num_doctor are the control variables; X represents all of the above core independent variables and control variables; W is the spatial weighting matrix; ε_it_ and μ_it_ are normally distributed random error vector; α denotes the intercept item; β denotes the influence coefficient of independent variables on dependent variable; ρ denotes the spatial autoregressive coefficient; λ denotes the spatial error coefficient; θ denotes the space lag coefficient of the independent variables; i represents regions, and t represents year.

#### 2.3.3. Model Test

In general, the Lagrange multiplier tests (i.e., LM-lag and LM-err) were used to determine a proper spatial econometric model [61], but these methods were only suitable for sectional data. For panel data, referring to the study of Belotti et al. [62], this study tested the conditions given in Table 3 to select the spatial econometric model.

In Table 3, the test results are all significant at the 1% level (*p* < 1), and we should reject these null assumptions that λ = 0 and λ = −*ρβ*. In other words, SDM cannot be simplified into SAR or SEM, and should be adopted to analyze the effect of PM_2.5_ exposure on residents’ health burden in China. Also, through the Hausman test (i.e., space fixed effect or time fixed effect), the result shows that the spatial econometric model should adopt fixed effect (*χ^2^* = 11.35, *p* = 0.0782 < 10%). From here, this study should use the SDM model with fixed effect to analyze.

## 3. Spatial Distribution and Spatial Autocorrelation Analysis

### 3.1. Spatial Distribution

**Figure 1:**
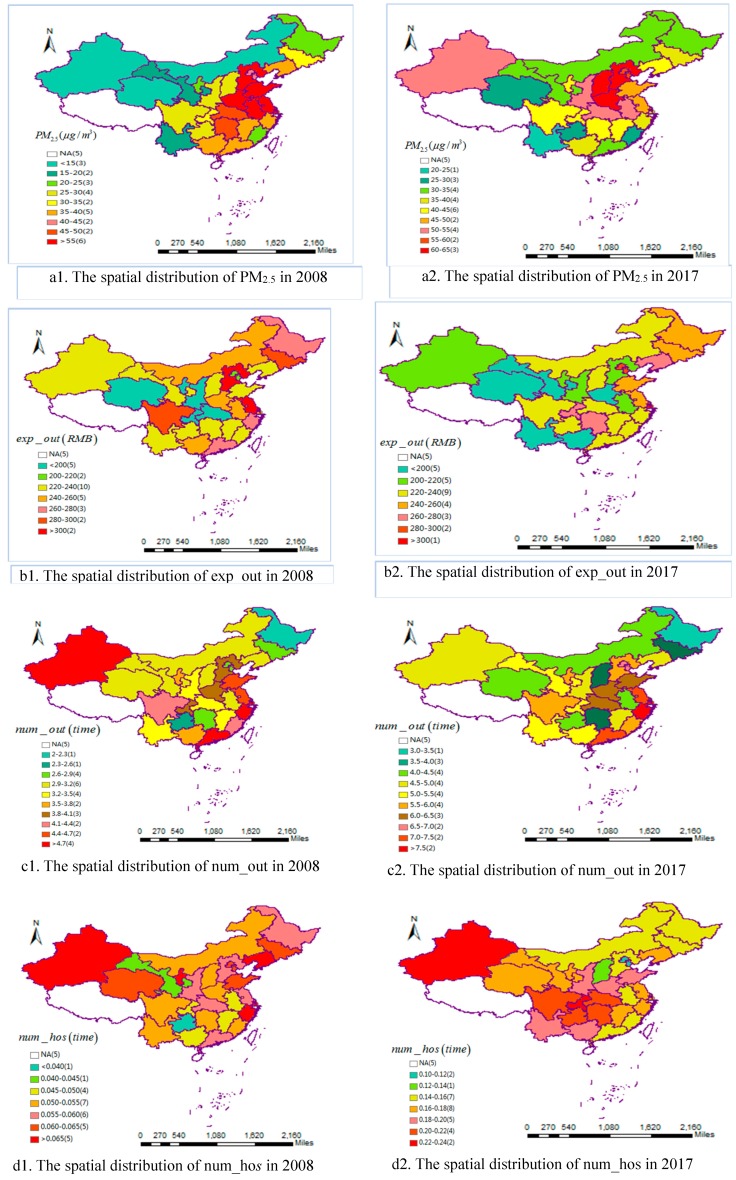
The spatial distribution of the core variables in 2008 and 2017.

Figure 1 shows the spatial distribution of PM_2.5_ concentrations (Figure 1(a1,a2)), outpatient expense (Figure 1(b1,b2)), outpatient visits (Figure 1(c1,c2)) and the number of hospitalization (Figure 1(d1,d2)) in all selected regions of China in 2008 and 2017. In absolute terms, compared with 2008, PM_2.5_ concentrations, outpatient visits and the number of hospitalization in different regions of China increased in 2017, whereas outpatient expense varied slightly. In relative terms, Chinese regions displayed similarities during the periods. For PM_2.5_ concentrations, outpatient visits and outpatient expense, regions with high-values were concentrated in the eastern districts, while regions with low-values were concentrated in the western and central districts. For the number of hospitalization, regions with high-values were concentrated in the western and central districts, and regions with low-values were concentrated in the eastern districts. Due to space limitation, we only chose the spatial distribution of PM_2.5_ concentrations, outpatient expense, outpatient visits and the number of hospitalizations in 2008 and 2017 to analyze.

From Figure 1(a2), we can see that PM_2.5_ pollution is the worst in Beijing, Hebei, Tianjin, and Henan, followed by Shaanxi, Hubei, Anhui, and Xinjiang. While the regions of southwest, southeast, and northeast in China, such as Yunnan, Qinghai, Guizhou, and Fujian, have the least pollution. What stands out in this figure is that PM_2.5_ concentration distribution in China has obvious spatial clustering characteristics. That is, the regions with the most serious PM_2.5_ pollution are concentrated together, and the regions with the least pollution are also concentrated together.

Figure 1(b2,c2,d2) present the distribution of residents’ health burden in all the selected regions of China. Similar to PM_2.5_ pollution, the distribution of residents’ health burden in China also shows obvious spatial clustering characteristics. Figure 1(b2,c2) show that outpatient expense and outpatient visits are positively correlated with the economic level in all different regions. For example, outpatient expense and outpatient visits in developed regions are significantly greater than those in backward regions. Besides, outpatient expense and outpatient visits are also significantly higher in regions with serious PM_2.5_ pollution than in regions with less pollution, such as Beijing, Shandong, Jiangsu, etc. However, for Figure 1(d2), the number of hospitalization per capita in western China is higher than that in eastern China. Since the medical treatment insurance system of the western regions in China is not perfect, some patients are hospitalized to get medical insurance compensation, even if they do not meet the standards of hospitalization, resulting in false hospitalization [63]. Because of the above shortages, this study chose outpatient expense and outpatient visits as the main independent variables.

### 3.2. Spatial Autocorrelation Analysis

Global Moran’s I of exp_out and PM_2.5_max_ based on the three spatial matrices from 2008 to 2017 were shown in Table 4. The results indicate that all the Moran’s I of exp_out and PM_2.5_max_ are significantly positive at the 5% level for the three spatial matrices, except that Moran’s I of PM_2.5_max_ are not all significant in the spatial economic matrix. These suggest that exp_out and PM_2.5_max_ are not randomly distributed in different regions of China, but have obvious spatial correlation and spatial clustering characteristics during the study period. In a word, the residents’ health expense and PM_2.5_ pollution in different regions of China are characterized by high-high (H-H) value aggregation and low-low (L-L) value aggregation. Therefore, the spatial correlation must be considered when studying the impact of PM_2.5_ exposure on health burden of residents in China, otherwise, the research results may be biased.

**Figure 2:**
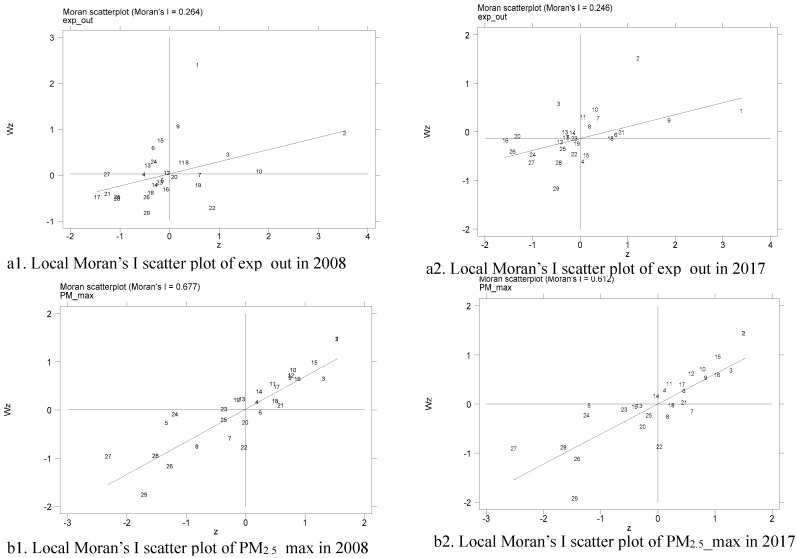
Local Moran’s I scatter plot in 2008 and 2017. Note: Numbers 1 to 29 represent Beijing, Tianjin, Hebei, Shanxi, Inner Mongolia, Liaoning, Jilin, Heilongjiang, Shanghai, Jiangsu, Zhejiang, Anhui, Fujian, Jiangxi, Shandong, Henan, Hubei, Hunan, Guangdong, Guangxi, Chongqing, Sichuan, Guizhou, Yunnan, Shaanxi, Gansu, Qinghai, Ningxia and Xinjiang, respectively.

To analyze the local agglomeration characteristics of all the selected regions in China, Figure 2 presents the local Moran’s I scatter plots of exp_out and PM_2.5__max based on spatial contiguity matrix W_1_ for all the regions in 2008 and 2017. In the local Moran’s I scatter plot, the horizontal axis represents the observations of the local region, and the vertical axis represents observations of the adjacent regions. In other words, the first quadrant and the third quadrant point out the existence of positive spatial correlation, representing high-high (H-H) value clustering and low-low (L-L) value clustering, respectively. While the second quadrant and the fourth quadrant mark the existence of negative spatial correlation, representing low-high (L-H) value clustering and high-low (H-L) value clustering, respectively.

As can be seen from Figure 2, for both 2008 and 2017, most of the regions are located in the first quadrant or the third quadrant, indicating that there is a positive spatial correlation between residents’ health expense and PM_2.5_ concentrations in most regions. This means that the residents’ health burden and PM_2.5_ concentrations of different regions in China are not random but show significantly positive spatial autocorrelation. For example, for the local Moran’s I scatter plots of exp_out in 2017, there are 9 regions located in the first quadrant, such as Beijing, Tianjin, Shanghai, Jiangsu, etc., and 10 regions are in the third quadrant, including Yunnan, Sichuan, Guizhou, Gansu, etc. The others are in the second or fourth quadrants. In the scatter plots of PM_2.5__max in 2017, there are 23 regions located in the first or third quadrants, such as Beijing, Tianjin, Hebei, Shanghai, etc. In a word, there is a positive spatial correlation between PM_2.5_ concentrations and residents’ health burden, which is consistent with the previous conclusion.

**Figure 3:**
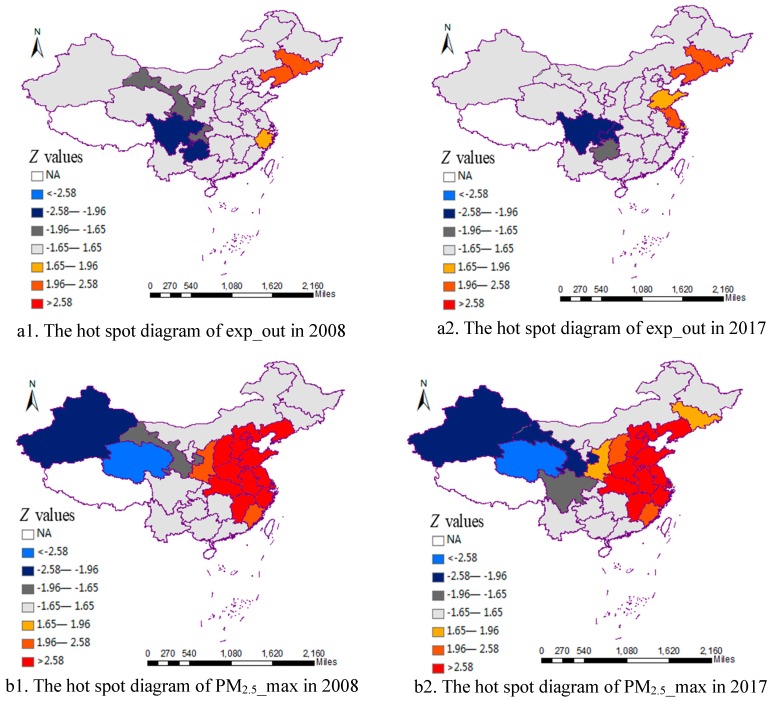
Hot spot analysis results in 2008 and 2017.

We also used hot spot analysis [64] to study the local agglomeration characteristics, and Figure 3 presents the analysis results of exp_out and PM_2.5__max for all the regions in China in 2008 and 2017. By calculating the Z value (namely Getis-Ord Gi*), we can identify hot spots and cold spots with statistical significance. If the absolute value of Z of the region is smaller than 1.65, it indicates that the region is less likely to be related to its neighboring regions. While if the absolute value is greater than 1.65, it indicates that there is a close connection among the regions [64]. Furthermore, the positive Z value indicates that the region is a hot spot, that is, the values of exp_out or PM_2.5__max of the region and its neighboring regions are all high. While the negative Z value indicates that the region is a cold spot, that is, the values of exp_out or PM_2.5__max of the region and its neighboring regions are all low. Meanwhile, 1.65, 1.96 and 2.58 are the threshold of 10%, 5%, and 1% significance level, respectively. As can be seen from Figure 3, for residents’ health expenses, Liaoning, Jilin, and Zhejiang are hot spots in 2008, and Liaoning, Jilin, Shangdong, and Jiangsu are hot spots in 2017. Gansu, Sichuan, Yunnan, and Chongqing are cold spots in 2008, and Sichuan, Yunnan, and Chongqing are cold spots in 2017. It can be seen that hot spots are distributed in the eastern districts, and cold spots in the western districts. For PM_2.5_ concentrations, there are a great number of hot spots and cold spots. Hot spots are mainly distributed in the Beijing–Tianjin–Hebei regions, central districts, southeast districts, Liaoning and Jilin, whereas cold spots are mainly distributed in northwest districts and Sichuan.

## 4. Empirical Analysis and Discussion

This study used SDM to explain the impact of PM_2.5_ exposure on residents’ health burden in China. In this section, we discussed the influence of PM_2.5_ exposure on outpatient expense and outpatient visits, respectively. According to the test results of the models, the fitting degree of the SDM under the space and time fixed effect is superior to other models, and the following tests are based on it (The results of SDM model with space-fixed effect and time-fixed effect were also tested. The SDM model based on the space-and-time fixed effect was superior to the above two models. Therefore, this study adopted the SDM model with space-and-time fixed effect for empirical testing).

### 4.1. Impact of PM_2.5_ Exposureon Outpatient Expense

The results based on the three spatial weight matrices for the whole sample were presented in Table 5. Among them, the column (1), column (3) and column (5) in Table 5 are the results of the impact of PM_2.5_ concentrations (PM_2.5_max_) on outpatient expense (exp_out) based on spatial contiguity matrix W_1_, spatial distance matrix W_2_ and spatial economy matrix W_3_, respectively. The column (2), column (4) and column (6) in Table 5 are the results of the influence of one stage lag of PM_2.5_ concentrations (PM_2.5_max_(−1)) on outpatient expense (exp_out) when spatial contiguity matrix W_1_, spatial distance matrix W_2_ and spatial economy matrix W_3_ are adopted, respectively.

First, when the three different spatial weight matrices are used, all these coefficients of PM_2.5_max_ are significantly positive at the 1% level. This shows that PM_2.5_ exposure will increase the outpatient expense of residents during the research period. Since exposure to air pollutants, such as PM_2.5_, causes health problems of residents, including increased incidence of respiratory and cardiovascular diseases, and then increases outpatient expense. Also, when the spatial contiguity matrix W_1_ and the spatial distance matrix W_2_ are used, the spatial lag coefficients of PM_2.5_max_ are also significantly positive at the 5% level. While the spatial lag coefficient of PM_2.5_max_ is positive, but it does not exceed the 10% significance level when the spatial economic matrix W_3_ is used. This suggests that the increase of PM_2.5_ concentrations in geographically close regions will cause an increase of outpatient expense, and increase the health burden of residents in a particular region. But the increase of PM_2.5_ concentrations in economically related regions has almost no impact on outpatient expense in a particular region. Therefore, the spatial effect of PM_2.5_ exposure on health burden is more influenced by neighboring or geographically close regions.

Second, the coefficients of PM_2.5_max_(−1) are positive at the 1% significance level when the three spatial weight matrices are used, respectively. This expresses indirectly that PM_2.5_ exposure has a long-term impact on residents’ health burden during the research period. Because long-term PM_2.5_ exposure can increase the incidence of chronic diseases such as cardiovascular disease, cancer, and diabetes [65,66,67]. As for the spatial lag of PM_2.5_max_(−1), the coefficients are positive at the 1% level and 5% level, respectively, when we use the spatial contiguity matrix W_1_ and the spatial distance matrix W_2_. While the coefficient is positive based on the spatial economic matrix W_3_, it does not pass the 10% significance level test. This points out that the increase of PM_2.5_ concentrations in neighboring or geographically close regions will promote the increase of outpatient expense in this region, while the increase of PM_2.5_ concentrations in economically similar regions will not have a significant impact on the outpatient expense in the local region.

Third, the spatial autoregressive coefficients *ρ* are significantly negative at the 1% and 10% levels, respectively, when the spatial distance matrix W_2_ and spatial economy matrix W_3_ are adopted. However, when we apply the spatial contiguity matrix W_1_, the spatial autoregressive coefficients are negative but do not pass the significant 10% level test. This suggests that there is a negative correlation of outpatient expense between a particular region and geographically or economically similar regions, and PM_2.5_ exposure has a spatial spillover effect on outpatient expense. That is to say, health expense in a particular region is not only affected by PM_2.5_ exposure in the region, but also influenced by outpatient expense in neighboring or economically connected regions. Therefore, if the spatial spillover effect and temporal lag effect are ignored, the research conclusion will be biased, and the impact of PM_2.5_ exposure on residents’ health burden will be greatly underestimated.

Finally, for all the control variables, the direct coefficients and the spatial lag coefficients of PGDP are significant negatives at the 10% level, suggesting that there is clear evidence for a negative correlation between PGDP and residents’ health burden. Moreover, with the improvement of economic level, the medical conditions will be improved and the possibility of getting sick is reduced to a certain extent. For the urbanization rate, the direct coefficients and the spatial lag coefficients are positive at the significant 10% level, indicating that urbanization in the region and its adjacent regions can significantly promote the increase of outpatient expense. As for the number of medical institutions on the impacts of outpatient expense, the direct coefficients are significantly negative at the 10% level based on the matrices W_1_ and W_2_, while the spatial lag coefficients are not robust. These results indicate that the increase in the number of medical institutions will increase the degree of competition, promote the institutions to improve the medical level and lower the medical price, and then make or become less the outpatient expense. For the number of hospital beds, the coefficients are all positive at significant 1% level, but the spatial lag coefficients are positive at significant 10% level when the matrix W_1_ is adopted. However, as for the number of doctors, the direct coefficients and the spatial lag coefficients have not passed the significant tests except for the spatial contiguity matrix W_1_.

Given the particularity of the spatial econometric model, we cannot directly find out the spatial spillover effect from the above models. This study calculated the direct effect, the spatial spillover effect and the total effect of PM_2.5_ concentrations on outpatient expense, respectively. The results were given in Table 6.

The direct effect, the spatial spillover effect and the total effect of PM_2.5_max_ and PM_2.5_max_(−1) are all significantly positive at the significance 5% level. Specifically, the direct effect value of PM_2.5_max_ is 0.0987, the spatial spillover effect value of PM_2.5_max_ is 0.1245, the total effect value of PM_2.5_max_ is 0.2232, and the spatial spillover effect accounts for about 55.78% of the total effect. This suggests that for every 1% increase in PM_2.5_ concentrations in the local region and adjacent regions, the outpatient expense will increase by approximately 9.87% and 12.45%, respectively. As for the PM_2.5_max_(−1), the direct effect value is 0.0942, the spatial spillover effect value of PM_2.5_max_ is 0.1283, the total effect value of PM_2.5_max_ is 0.2225, and the spatial spillover effect accounts for about 57.66% of the total effect. This points out that every 1% increase in PM_2.5_ concentrations in a particular region and adjacent regions, outpatient expense will increase about 9.42% and 12.83%, respectively. In other words, the influence of PM_2.5_ exposure on outpatient expense in China is mainly due to spatial spillover effect. This further proves that the spatial spillover effects of PM_2.5_ exposure on health burden cannot be ignored.

### 4.2. Impact of PM_2.5_ Exposure on Outpatient Visits

As the health burden of residents is a comprehensive concept, this study adopted another variable, outpatient visits, to measure the health burden of residents. The results of the impact of PM_2.5_ exposure on outpatient visits were presented in Table 7. Among them, the column (1), column (3) and column (5) in Table 7 are the results of PM_2.5_ exposure (PM_2.5__max) on outpatient visits (num_out) based on the three spatial matrices from W_1_ to W_3_. The column (2), column (4) and column (6) in Table 7 are the results of the influence of one stage lag of PM_2.5_ exposure (PM_2.5__max(−1)) on outpatient visits (num_out) when the spatial weight matrices change from W_1_ to W_3_.

First, the direct coefficients of PM_2.5__max and PM_2.5__max(−1) are significantly positive at the 1% level when the spatial contiguity matrix W_1_ and spatial distance matrix W_2_ are used. While the coefficients are positive but not significant at 10% level based on the spatial economy matrix W_3_. This indicates that PM_2.5_ exposure has a positive impact on outpatient visits, and the influence of PM_2.5_ exposure on outpatient visits has a temporal lag effect. The main reason is that, with the increase of PM_2.5_ concentration, the incidence of acute and chronic diseases such as respiratory system diseases and cardiovascular diseases will increase, which leads to an increase in outpatient visits.

Second, as for the spatial lag of PM_2.5_max_ and PM_2.5_max_(−1), the coefficients are all negative at the 1% significance level when the spatial weight matrices change from W_1_ to W_3_, showing that there is a negative spatial spillover effect of PM_2.5_max_ and PM_2.5_max_(−1) on outpatient visits in China. In other words, whether in the short or long term, the increase of PM_2.5_ concentrations in geographically close or economically similar regions will promote the decrease of outpatient visits in a particular region. These results are different from the spatial impact of PM_2.5_ exposure on outpatient expense discussed above.

Third, the spatial autoregressive coefficients *ρ* are significantly positive at the 1% level when the spatial matrix is W_1_, and significantly negative at the 1% level based on the spatial economy matrix W_3_. However, they do not pass the significant test when the spatial matrix is W_2_. This proves that the outpatient visits of adjacent regions have a positive influence on that of the particular region, while there is a negative correlation of outpatient visits between the particular region and economically similar regions. Therefore, spatial spillover effect should be considered when analyzing the impact of PM_2.5_ exposure on health burden of residents.

We also calculated the direct effect, the spatial spillover effect and the total effect of PM_2.5_ concentrations on outpatient visits of residents, respectively, and the results are shown in Table 8. The results suggest that the direct effect of PM_2.5_max_ and PM_2.5_max_(−1) are significantly positive at the 1% level, while both the spatial spillover effect and the total effect are significantly negative at the significance level of 5%. These results are consistent with the above conclusions.

For the coefficient value of PM_2.5_max_, the direct effect value is 0.1944, the spatial spillover effect value is −0.3516, and the total effect value is −0.1572. The spatial spillover effect of PM_2.5_max_ is much larger than the direct effect. This indicates that for every 1% increase in PM_2.5_ concentrations in a particular region and adjacent regions, it will lead to an increase of about 19.44% and a decrease of 35.16% in outpatient visits in the particular region, respectively.

As for the PM_2.5_max_(−1), the direct effect value is 0.1984, the spatial spillover effect value is −0.3497, and the total effect value is −0.1513. The value of the spatial spillover effect is also larger than that of the direct effect. The conclusions further confirm that PM_2.5_ exposure has an important spatial spillover effect on residents’ health burden. It also points out that if the spatial models are not adopted, the direct impact of PM_2.5_ exposure may be overestimated and the spatial spillover effect may be underestimated.

### 4.3. Robustness Tests

#### 4.3.1. Alternative Independent Variable Estimation

This study took the maximum value of PM_2.5_ concentrations as the core independent variable to analyze the spatial impact of PM_2.5_ exposure on residents’ health burden. To avoid the selection bias of the independent variable, the mean value of PM_2.5_ concentrations were selected as the substitution variable of PM_2.5_ exposure for the robustness test. The results were given in column (1) and column (2) of Table 9.

The results show that the direct coefficient and the spatial lag coefficient of PM_2.5__avg are significantly positive at the 1% level when the spatial matrix is W_1_. As for the PM_2.5__avg(−1), the direct coefficient and the spatial lag coefficient are also significantly positive at 5% level based on the spatial contiguity matrix W_1_. This suggests that the average of PM_2.5_ concentrations in a particular region or the adjacent regions has a positive impact on outpatient expense in the particular region, with spatial spillover effect and temporal lag effect. The results are consistent with the above conclusions, indicating that the results are stable and reliable. In other words, PM_2.5_ exposure has temporal lag effect and spatial spillover effect on residents’ health burden.

#### 4.3.2. Alternative Dependent Variable Estimation

In this study, outpatient visits and outpatient expenses were selected as the substitution variables of health burden. To eliminate bias in the selection of indicators, this study used the number of hospitalizations (num_hos) as the dependent variable for robustness tests. The results were given in column (3) and column (4) of Table 9. The direct coefficients of PM_2.5_max_ and PM_2.5_max_(−1) are all significant positive at 1% level when the spatial matrix is W_1_, while the spatial lag coefficients are significantly negative at 1% level. The results are consistent with the above conclusions and are robust, suggesting that PM_2.5_ exposure has spatial spillover effect and temporal lag effect on residents’ health burden.

#### 4.3.3. Endogenous Test

Due to the two-way influence between the dependent variable and the independent variable or some important omitted variables, there may be endogenous problems between PM_2.5_ exposure and health burden. To reduce the possibility of estimation errors caused by endogenous problems, this study used the spatial Generalized Method of Moments (GMM) [60] to verify the reliability of the main empirical results. Kelejian et al. [68] proposed that $W{\left(1-\lambda W\right)}^{-1}X\beta$ was the relatively ideal instrumental variable, but the value of *λ* cannot be obtained in advance. Referring to the study of Yu and Liu [69], W*PM_2.5_max_ and W*PM_2.5_max_(−1) were selected as the instrumental variables of the spatial GMM method, and Hansen J test was used to verify the rationality of the selected instrumental variables.

The results of the spatial GMM method were presented in column (5) and column (6) of Table 9. As can be seen from the results, the *p* values of Hansen J test are 0.2952 and 0.2276, respectively, indicating that W*PM_2.5_max_ and W*PM_2.5_max_(−1) are suitable as instrumental variables. The coefficients are all significant positive at the 1% level, indicating that the results of the spatial GMM estimation are consistent with the previous results. Therefore, the results of this paper are robust and reliable.

## 5. Conclusions

Using the panel data of 29 Chinese regions from 2007 to 2017, this study used the spatial Durbin model (SDM) under space and time fixed effect to estimate the direct and the spatial lag effects of PM_2.5_ exposure on residents’ health burden in China based on three representative spatial weight matrices. The main conclusions drawn were as follows:

(1) Residents’ health burden and PM_2.5_ exposure are not randomly distributed among different regions in China, but have obvious spatial correlation and spatial clustering characteristics. There is a positive spatial correlation between PM_2.5_ concentrations and residents’ health burden. Also, the health burden in a particular region is not only affected by PM_2.5_ exposure in this region, but also influenced by the health burden in neighboring or economically similar regions.

(2) PM_2.5_ exposure has a significant positive impact on the health burden of residents in China. For example, PM_2.5_ pollution will increase outpatient expense and outpatient visits of residents. The possible reason is that exposure to air pollutants, such as PM_2.5_, makes the incidence of acute and chronic diseases such as respiratory diseases and cardiovascular diseases increase, and then the outpatient expense and outpatient visits will also increase.

(3) PM_2.5_ exposure has a spatial spillover effect on health burden. The increase of PM_2.5_ concentrations in surrounding regions or geographically close regions will lead to an increase of outpatient expense, but reduce outpatient visits in a particular region. Moreover, the value of the spatial spillover effect is larger than that of the direct effect, and the influence of PM_2.5_ exposure on health burden in China is mainly due to the spatial spillover effect.

(4) PM_2.5_ exposure has a long-term impact on residents’ health burden, that is to say, it has a temporal lag effect. Health burden in a particular region is not only affected by PM_2.5_ exposure in this region for a long time, but also affected by PM_2.5_ exposure in adjacent or geographically close regions for a long time. Long-term PM_2.5_ exposure can increase the incidence of chronic diseases such as cardiovascular disease, cancer, and diabetes.

According to the conclusions above, we have drawn some policy implications as follows:

(1) The impact of PM_2.5_ exposure on the health burden of residents in China must be given attention in the future. Frequent exposure to PM_2.5_ pollution can cause many acute and chronic diseases, which, eventually, brings great economic burden to residents. Hence, the government should strengthen environmental inspection and punishment, and shut down the enterprises with high pollution and low efficiency.

(2) Governments at all levels should break the administrative monopolies and achieve cross-regional cooperation in the field of environmental protection. The influence of PM_2.5_ exposure on health burden in China is mainly due to the spatial spillover effect, and this non-negligible fact urgently requires governments at all levels to strengthen the sense of cooperation, share information and technology of pollution control, and establish a common environmental protection system.

(3) Governments should establish a warning system and a long-term governance mechanism for environmental pollution. The impact of PM_2.5_ exposure on health burden is a long-term process, which can be easily overlooked. Therefore, the government should increase environmental protection publicity and improve residents’ awareness of environmental protection, so as to reduce residents health damage caused by environmental pollution. 

## Figures and Tables

**Table 1:** Description of the variable.

Type	Variable	Symbol	Definition
Dependent variable	Outpatient expense	exp_out	The ratio of the total outpatient expense to the total number of outpatient visits in the form of the natural logarithm
	Outpatient visits	num_out	The ratio of the total number of outpatient visits to the total population in the form of the natural logarithm
	The number of hospitalization	num_hos	The ratio of the total number of hospitalization to the total population
Independent variable	Maximum PM_2.5_ concentrations	PM_2.5_max_	The maximum values of PM_2.5_ concentrations in the form of natural logarithm
	Maximum PM_2.5_ concentrations lag by one stage	PM_2.5_max_(−1)	The maximum values of the last year’s PM_2.5_ concentrations in the form of the natural logarithm
	Average PM_2.5_ concentrations	PM_2.5_avg_	The average values of PM_2.5_ concentrations in the form of the natural logarithm
	Average PM_2.5_ concentrations lag by one stage	PM_2.5_avg_(−1)	The average values of the last year’s PM_2.5_ concentrations in the form of natural logarithm
Control variable	Per capita GDP	PGDP	The ratio of gross domestic product to the total population in the form of the natural logarithm
	The ratio of urban population	urban	The ratio of the urban population to the total population
	The number of medical institutions	num_inst	The ratio of the total number of medical institutions to the total population in the form of the natural logarithm
	The number of hospital beds	num_bed	The ratio of the total number of hospital beds to the total population in the form of the natural logarithm
	The number of doctors	num_doctor	The ratio of the total number of doctors to the total population in the form of the natural logarithm

**Table 2:** Descriptive statistics.

Variable	Obs	Mean	S.D.	Min	Median	Max
exp_out	290	5.230	0.297	4.385	5.242	6.248
num_out	290	1.548	0.324	0.832	1.501	2.397
num_hos	290	0.125	0.043	0.039	0.126	0.224
PM_2.5_max_	290	3.847	0.413	2.605	3.903	4.575
PM_2.5_max_(−1)	290	3.841	0.414	2.605	3.897	4.575
PM_2.5_avg_	290	3.446	0.534	1.938	3.519	4.404
PM_2.5_avg_(−1)	290	3.422	0.549	1.938	3.488	4.404
PGDP	290	1.368	0.514	−0.010	1.369	2.557
urban	290	0.548	0.134	0.291	0.530	0.896
num_inst	290	1.786	0.510	0.208	1.949	2.455
num_bed	290	3.773	0.236	3.140	3.802	4.227
num_doctor	290	4.293	0.198	3.689	4.310	4.978

**Table 3:** The selection results of spatial autoregression model (SAR), spatial errors model (SEM), and spatial Durbin model (SDM).

Name	Model	Selection Criteria	Chi-Square Value	*p*-Value
SAR	y=ρWy+Xβ+ε	λ=0	32.32	0.0000
SEM	y=Xβ+u,u=λWu+ε	λ=−ρβ	31.37	0.0000
SDM	y=ρWy+Xβ+λWX+ε	λ≠0&λ≠−ρβ		

Hausman test: The Chi-square value is 11.35, and the *p*-value is 0.0782.

**Table 4:** Global Moran’s I values of exp_out and PM_2.5_max_ (2008–2017).

Year	exp_out			PM_2.5__max		
	W_1_	W_2_	W_3_	W_1_	W_2_	W_3_
2008	0.201 **	0.058 **	0.079 *	0.527 ***	0.238 ***	0.097 *
2009	0.278 ***	0.167 ***	0.307 ***	0.519 ***	0.243 ***	0.091 *
2010	0.270 ***	0.168 ***	0.296 ***	0.514 ***	0.233 ***	0.080
2011	0.256 ***	0.163 ***	0.339 ***	0.504 ***	0.236 ***	0.067
2012	0.227 ***	0.149 ***	0.310 ***	0.511 ***	0.225 ***	0.038
2013	0.215 ***	0.136 ***	0.267 ***	0.512 ***	0.256 ***	0.062
2014	0.197 ***	0.122 ***	0.254 ***	0.542 ***	0.242 ***	0.064
2015	0.170 ***	0.101 ***	0.234 ***	0.525 ***	0.261 ***	0.112 *
2016	0.167 ***	0.102 ***	0.245 ***	0.545 ***	0.289 ***	0.081
2017	0.163 **	0.091 ***	0.245 ***	0.468 ***	0.239 ***	0.099 *

Note: ***, **, and * denote significance at the 1%, 5%, and 10% levels, respectively; The Moran’s I values of other variables are not reported for space limitation.

**Table 5:** Estimation results of the impact of PM_2.5_ exposure on outpatient expense.

Variable	Spatial Contiguity Matrix W_1_		Spatial Distance Matrix W_2_		Spatial Economy Matrix W_3_	
	(1)	(2)	(3)	(4)	(5)	(6)
PM_2.5_max_	0.1017 ***		0.1282 ***		0.1773 ***	
	(2.79)		(4.41)		(8.58)	
PM_2.5_max_(−1)		0.0971 ***		0.1186 ***		0.1713 ***
		(2.65)		(4.16)		(8.34)
PGDP	−0.2317 ***	−0.2323 ***	−0.3029 ***	−0.3097 ***	−0.1916 ***	−0.1975 ***
	(−4.76)	(−4.76)	(−5.39)	(−5.51)	(−2.70)	(−2.77)
urban	1.3876 ***	1.3840 ***	1.7333 ***	1.7686 ***	1.2335 ***	1.2656 ***
	(7.32)	(7.30)	(10.04)	(10.22)	(6.86)	(7.03)
num_inst	−0.0710 **	−0.0717 **	−0.0469 *	−0.0449 *	−0.0226	−0.0191
	(−2.47)	(−2.49)	(−1.76)	(−1.68)	(−0.78)	(−0.66)
num_bed	0.4853 ***	0.4929 ***	0.3549 ***	0.3659***	0.3868 ***	0.3915 ***
	(5.76)	(5.85)	(5.29)	(5.47)	(5.57)	(5.58)
num_doctor	0.0178	0.0118	0.1052	0.0887	−0.0487	−0.0556
	(0.23)	(0.15)	(1.26)	(1.06)	(−0.57)	(−0.64)
W*PM_2.5_max_	0.1531 ***		0.4066 **		0.0106	
	(2.76)		(2.12)		(0.17)	
W*PM_2.5_max_(−1)		0.1551 ***		0.4771 **		0.0012
		(2.78)		(2.55)		(0.02)
W*PGDP	−0.4213 ***	−0.4178 ***	−1.4444 ***	−1.4888 ***	−0.0383	−0.0382
	(−4.62)	(−4.58)	(−4.70)	(−4.84)	(−0.31)	(−0.31)
W*urban	2.7245 ***	2.7882 ***	5.4106 ***	5.6841 ***	0.7962	0.8438 *
	(6.62)	(6.77)	(4.43)	(4.66)	(1.56)	(1.65)
W*num_inst	0.1294 *	0.1458 **	−0.3039	−0.2645	−0.3431 ***	−0.3369 ***
	(1.87)	(2.08)	(−1.23)	(−1.07)	(−4.05)	(−3.95)
W*num_bed	0.3319 *	0.3426 **	0.2244	0.3220	−0.0646	−0.0803
	(1.91)	(1.96)	(0.47)	(0.68)	(−0.30)	(−0.37)
W*num_doctor	−0.7153 ***	−0.7542 ***	0.0433	−0.0844	−0.0392	−0.0449
	(−3.55)	(−3.71)	(0.08)	(−0.15)	(−0.15)	(−0.17)
*ρ*	−0.1312	−0.1230	−0.7074 ***	−0.7217 ***	−0.2088 *	−0.2034 *
	−1.49)	(−1.40)	(−2.95)	(−3.01)	(−1.78)	(−1.73)
sigma2_e	0.0122 ***	0.0123 ***	0.0118 ***	0.0118 ***	0.0123 ***	0.0124 ***
	(11.94)	(11.95)	(12.03)	(12.03)	(12.30)	(12.29)
*N*	290	290	290	290	290	290

Notes: ***, **, and * represent significance at the 1%, 5% and 10% levels, respectively; The numbers in brackets are t statistic values.

**Table 6:** The direct effects, the spatial spillover effects and the total effects of SDM (the dependent variable is exp_out).

Type	Variable	Coefficient	t−Value	*p*−Value
Direct effects	PM_2.5_max_	0.0987 **	2.55	0.011
	PM_2.5_max_(−1)	0.0942 **	2.42	0.015
Spatial Spillover Effects	PM_2.5_max_	0.1245 **	2.37	0.018
	PM_2.5_max_(−1)	0.1283 **	2.42	0.016
Total Effects	PM_2.5_max_	0.2232 ***	7.11	0.000
	PM_2.5_max_(−1)	0.2225 ***	7.02	0.000

Note: *** and ** represent significance at the 1% and 5% levels, respectively.

**Table 7:** The results of the spatial impact of PM_2.5_ exposure on outpatient visits.

Variable	Spatial Contiguity Matrix W_1_		Spatial Distance Matrix W_2_		Spatial Economy Matrix W_3_	
	(1)	(2)	(3)	(4)	(5)	(6)
PM_2.5_max_	0.2114 ***		0.3311 ***		0.0070	
	(4.10)		(8.56)		(0.24)	
PM_2.5_max_(−1)		0.2154 ***		0.3169 ***		0.0178
		(4.16)		(8.22)		(0.61)
PGDP	0.1840 ***	0.1874 ***	0.3900 ***	0.3944 ***	0.3556 ***	0.3569 ***
	(2.74)	(2.79)	(5.25)	(5.23)	(3.50)	(3.52)
urban	0.6517 **	0.6314 **	−0.4861 **	−0.5139 **	0.2340	0.2304
	(2.54)	(2.46)	(−2.13)	(−2.22)	(0.90)	(0.90)
num_inst	−0.0963 **	−0.1007 **	−0.2164 ***	−0.2206 ***	−0.1941 ***	−0.1933 ***
	(−2.40)	(−2.52)	(−6.12)	(−6.16)	(−4.68)	(−4.68)
num_bed	−0.7171 ***	−0.7150 ***	−0.6721 ***	−0.6841 ***	−0.4667 ***	−0.4541 ***
	(−6.10)	(−6.09)	(−7.50)	(−7.54)	(−4.70)	(−4.57)
num_doctor	0.8027 ***	0.8038 ***	0.9740 ***	0.9828 ***	0.4070 ***	0.3994 ***
	(7.65)	(7.65)	(8.75)	(8.71)	(3.34)	(3.28)
W*PM_2.5_max_	−0.3241 ***		−2.3216 ***		−0.2375 ***	
	(−4.33)		(−9.33)		(−2.66)	
W*PM_2.5_max_(−1)		−0.3232 ***		−2.1647 ***		−0.2460 ***
		(−4.29)		(−8.76)		(−2.77)
W*PGDP	−0.1371	−0.1416	0.6240	0.6221	−0.3989 **	−0.3803 **
	(−1.11)	(−1.15)	(1.53)	(1.50)	(−2.21)	(−2.11)
W*urban	−1.7539 ***	−1.7123 ***	−2.6430	−2.9903 *	1.9654 ***	1.8389 ***
	(−3.23)	(−3.16)	(−1.62)	(−1.83)	(2.78)	(2.61)
W*num_inst	−0.5490 ***	−0.5367 ***	−0.8454 **	−0.8419 **	−0.3412 ***	−0.3607 ***
	(−5.05)	(−4.90)	(−2.47)	(−2.42)	(−2.83)	(−2.99)
W*num_bed	−0.1504	−0.1298	−3.0001 ***	−2.7721 ***	0.3599	0.3428
	(−0.59)	(−0.51)	(−4.66)	(−4.27)	(1.17)	(1.12)
W*num_doctor	0.6217 **	0.5846 *	2.2185 ***	2.1068 ***	−1.2269 ***	−1.2073 ***
	(1.96)	(1.83)	(2.89)	(2.69)	(−3.29)	(−3.26)
*ρ*	0.2720 ***	0.2758 ***	0.1090	0.1156	−0.2840 **	−0.2862 **
	(3.51)	(3.56)	(0.56)	(0.59)	(−2.36)	(−2.38)
sigma2_e	0.0238 ***	0.0238 ***	0.0208 ***	0.0213 ***	0.0252 ***	0.0251 ***
	(11.95)	(11.93)	(12.08)	(12.09)	(12.13)	(12.14)
*N*	290	290	290	290	290	290

Note: ***, **, and * represent significance at the 1%, 5% and 10% levels, respectively; The numbers in brackets are t statistic values.

**Table 8:** The direct effects, the spatial spillover effects and the total effects of SDM (the dependent variable is num_out).

Type	Variable	Coefficient	t-Value	*p*-Value
Direct Effects	PM_2.5_max_	0.1944 ***	3.92	0.000
	PM_2.5_max_(−1)	0.1984 ***	3.99	0.000
Spatial Spillover Effects	PM_2.5_max_	−0.3516 ***	−4.09	0.000
	PM_2.5_max_(−1)	−0.3497 ***	−4.03	0.000
Total Effects	PM_2.5_max_	−0.1572 **	−2.26	0.024
	PM_2.5_max_(−1)	−0.1513 **	−2.16	0.031

Note: *** and ** represent significance at the 1% and 5% level, respectively.

**Table 9:** Results of the robustness tests.

Variable	exp_out	exp_out	num_hos	num_hos	GMM	GMM
	(1)	(2)	(3)	(4)	(5)	(6)
PM_2.5_max_			0.0335 ***			
			(8.60)			
PM_2.5_max_(−1)				0.0329 ***		
				(8.33)		
PM_2.5_avg_	0.0603 ***					
	(3.16)					
PM_2.5_avg_(−1)		0.0607 ***				
		(3.35)				
PGDP	−0.3068 ***	−0.3404 ***	0.0141 ***	0.0143 ***	1.3684 ***	1.3684 ***
	(−5.21)	(−5.81)	(2.73)	(2.76)	(45.35)	(45.35)
urban	1.7402 ***	1.8307 ***	−0.0923 ***	−0.0956 ***	0.5480 ***	0.5480 ***
	(9.64)	(10.21)	(−4.61)	(−4.74)	(69.55)	(69.55)
num_inst	−0.0421	−0.0398	−0.0112 ***	−0.0119 ***	1.7864 ***	1.7864 ***
	(−1.48)	(−1.42)	(−3.66)	(−3.86)	(59.62)	(59.62)
num_bed	0.3559 ***	0.3656 ***	0.0985 ***	0.0997 ***	3.7732 ***	3.7732 ***
	(5.36)	(5.23)	(11.01)	(11.07)	(271.75)	(271.75)
num_doctor	0.1363	0.1422 *	0.0051	0.0053	4.2932 ***	4.2932 ***
	(1.55)	(1.64)	(0.63)	(0.65)	(370.05)	(370.05)
W*PM_2.5_max_			−0.0484 ***		3.8471 ***	
			(−8.44)		(158.76)	
W*PM_2.5_max_(−1)				−0.0472 ***		3.8407 ***
				(−8.11)		(157.95)
W*PM_2.5_avg_	0.2953 **					
	(2.18)					
W*PM_2.5_avg_(−1)		0.4607 ***				
		(3.31)				
W*PGDP	−1.3705 ***	−1.6526 ***	−0.0333 ***	−0.0341 ***		
	(−4.18)	(−4.94)	(−3.49)	(−3.55)		
W*urban	5.3744 ***	5.8229 ***	0.1284 ***	0.1368 ***		
	(4.27)	(4.68)	(3.09)	(3.28)		
W*num_inst	−0.3455	−0.3123	−0.0004	0.0010		
	(−1.34)	(−1.23)	(−0.05)	(0.12)		
W*num_bed	−0.3486	−0.0944	−0.0570 ***	−0.0556 ***		
	(−0.75)	(−0.20)	(−3.04)	(−2.92)		
W*num_doctor	0.4873	0.5663	−0.0430 **	−0.0472 **		
	(0.83)	(0.97)	(−2.00)	(−2.17)		
*ρ*	−0.5548 **	−0.6443 ***	0.6150 ***	0.6131 ***		
	(−2.38)	(−2.72)	(11.86)	(11.75)		
sigma2_e	0.0132 ***	0.0128 ***	0.0001 ***	0.0001 ***		
	(12.04)	(12.03)	(11.57)	(11.58)		
*N*	290	290	290	290	290	290

Notes: ***, **, and * represent significance at the 1%, 5% and 10% level, respectively; The numbers in brackets are t statistic values.

## Appendix A

**Table A1:** Average PM_2.5_ concentrations of 29 regions in China from 2007 to 2017 (μg/m^3^).

Region	2007	2008	2009	2010	2011	2012	2013	2014	2015	2016	2017
Beijing	50.81	48.21	47.35	48.59	43.02	48.06	41.55	50.00	44.06	48.46	45.00
Tianjin	81.93	74.84	74.95	78.14	70.81	71.88	62.03	81.79	71.47	74.82	70.79
Hebei	62.95	60.32	54.69	57.14	52.64	53.13	49.84	61.00	53.52	55.35	55.99
Shanxi	33.98	33.93	26.71	27.76	26.56	27.11	24.82	30.08	25.37	25.92	24.97
Inner Mongolia	11.07	12.28	11.92	11.77	12.04	10.52	10.12	21.05	10.88	13.52	12.49
Liaoning	33.02	34.49	37.19	38.34	35.85	33.13	28.49	35.81	34.47	47.66	34.61
Jilin	28.82	29.79	32.65	34.62	32.59	29.42	26.24	33.24	32.12	47.53	34.47
Heilongjiang	19.23	18.36	20.39	21.83	21.59	18.42	16.83	22.09	22.49	32.68	26.69
Shanghai	52.07	56.95	56.78	58.01	51.61	49.96	44.70	54.15	47.31	61.08	50.85
Jiangsu	61.23	61.74	59.58	60.00	59.97	58.06	50.20	60.65	57.22	65.39	58.31
Zhejiang	33.38	37.86	38.35	34.29	33.81	31.62	31.70	34.90	34.27	33.21	28.58
Anhui	49.57	58.11	55.19	52.24	53.38	49.67	45.46	53.13	53.81	57.02	46.15
Fujian	23.73	24.65	23.23	21.74	20.68	19.96	19.56	20.37	21.29	19.91	20.00
Jiangxi	37.63	41.04	39.74	37.46	36.72	33.67	34.56	34.93	37.99	34.86	31.36
Shandong	64.44	69.31	60.95	58.24	64.12	57.36	55.35	64.77	57.81	61.65	62.53
Henan	60.31	65.44	50.66	50.87	54.51	52.10	48.74	61.33	51.56	52.56	48.91
Hubei	45.82	49.18	46.88	45.58	49.40	45.47	40.35	46.29	48.14	47.29	37.68
Hunan	41.63	46.79	45.02	43.05	40.58	37.99	39.47	37.93	40.88	36.55	31.43
Guangdong	31.33	34.20	35.28	34.32	30.74	29.13	28.60	28.93	33.49	26.75	25.49
Guangxi	35.25	38.76	38.20	37.71	33.92	34.51	36.17	35.08	36.97	29.95	28.67
Chongqing	39.01	36.18	32.13	32.30	35.43	30.37	30.77	30.94	28.98	25.90	23.28
Sichuan	37.16	29.48	29.71	28.39	34.60	30.00	29.68	31.11	28.53	23.14	22.85
Guizhou	29.93	29.19	29.71	29.98	28.55	28.81	28.77	26.41	28.93	23.14	20.74
Yunnan	16.27	16.09	16.37	16.61	16.57	17.62	15.75	18.07	17.26	14.77	14.25
Shaanxi	32.31	32.74	25.68	27.36	28.21	28.07	26.32	31.82	25.76	26.28	24.23
Gansu	21.39	22.11	19.39	18.22	18.44	17.71	16.32	21.05	18.34	15.15	15.38
Qinghai	9.71	9.93	10.04	9.10	10.92	8.70	8.16	10.65	9.70	6.94	7.85
Ningxia	24.02	20.85	20.51	19.92	21.03	17.34	16.91	21.94	19.60	17.32	17.30
Xinjiang	9.04	7.78	9.06	8.39	8.69	7.70	7.75	9.77	8.72	10.43	11.50

**Table A2:** Maximum PM_2.5_ concentrations of 29 regions in China from 2007 to 2017 (μg/m^3^).

Region	2007	2008	2009	2010	2011	2012	2013	2014	2015	2016	2017
Beijing	102.10	90.80	88.70	89.70	85.60	92.00	78.60	97.00	84.30	93.50	88.90
Tianjin	96.10	89.00	88.50	90.80	83.80	87.00	75.00	96.20	86.10	84.60	84.10
Hebei	90.24	86.91	80.84	84.26	78.54	80.29	74.94	88.25	81.16	81.97	84.65
Shanxi	62.63	60.25	51.49	53.26	51.29	51.77	49.26	55.43	50.45	50.35	51.29
Inner Mongolia	26.68	27.57	27.87	27.56	27.60	25.08	24.53	30.06	26.62	32.67	29.68
Liaoning	48.08	49.34	52.65	54.36	51.15	48.95	42.80	51.18	49.37	63.48	50.55
Jilin	38.97	40.83	42.83	46.27	43.71	39.90	36.43	44.84	43.83	67.19	47.13
Heilongjiang	34.24	33.18	34.22	38.81	37.29	33.05	30.92	38.75	38.95	56.52	43.22
Shanghai	60.10	65.80	64.40	65.20	56.60	57.50	52.70	62.70	57.90	73.90	59.50
Jiangsu	67.92	68.58	65.76	65.92	66.45	64.05	55.94	67.04	63.93	72.25	64.88
Zhejiang	51.66	56.85	57.27	53.29	51.46	49.53	50.04	54.35	52.73	52.25	47.15
Anhui	59.15	68.31	64.88	62.39	63.00	58.51	54.29	63.08	65.01	66.88	55.85
Fujian	47.62	48.24	46.49	44.93	43.87	43.22	42.37	43.88	45.06	43.87	43.70
Jiangxi	50.33	54.01	52.22	50.61	49.77	45.95	47.21	47.91	51.87	48.44	44.33
Shandong	80.28	84.74	75.99	72.74	79.43	72.10	71.08	80.45	73.63	77.12	79.54
Henan	78.56	83.93	67.74	68.16	72.04	69.85	65.70	80.07	70.34	70.09	67.53
Hubei	57.63	61.85	58.80	57.47	62.58	57.44	51.22	59.29	61.58	60.62	49.24
Hunan	53.39	58.99	58.15	55.76	53.34	50.07	51.76	50.86	55.29	49.62	43.48
Guangdong	41.08	44.34	44.86	44.23	40.25	38.04	37.79	37.96	43.83	35.56	34.21
Guangxi	44.74	48.71	47.54	47.47	42.94	43.33	45.32	44.45	45.89	38.84	37.56
Chongqing	72.50	72.20	60.50	58.40	63.60	57.40	57.70	59.10	55.60	51.70	44.70
Sichuan	57.32	47.46	47.19	45.56	53.48	47.53	46.91	49.98	46.19	39.77	39.54
Guizhou	40.97	40.32	41.16	41.81	40.14	40.36	40.77	37.42	40.62	34.52	31.49
Yunnan	29.53	29.73	29.51	29.99	30.51	31.43	29.32	32.10	31.05	28.14	27.66
Shaanxi	50.02	50.38	41.12	42.99	43.96	44.20	41.20	49.68	42.09	42.64	40.87
Gansu	30.59	32.34	28.46	26.22	26.31	25.83	23.49	30.06	26.76	22.36	23.14
Qinghai	17.73	18.75	18.75	16.73	19.24	15.79	15.28	19.31	17.66	13.54	15.66
Ningxia	29.60	26.06	25.86	24.82	26.06	22.06	21.98	27.32	24.58	22.68	23.08
Xinjiang	25.44	22.86	23.91	23.31	23.66	21.28	21.36	25.56	23.67	26.52	29.57
